# Post-Processing PEEK 3D-Printed Parts: Experimental Investigation of Annealing on Microscale and Macroscale Properties

**DOI:** 10.3390/polym17060744

**Published:** 2025-03-12

**Authors:** Makenzie Adamson, Babak Eslami

**Affiliations:** Mechanical Engineering Department, Widener University, One University Place, Chester, PA 19013, USA; mgadamson@widener.edu

**Keywords:** PEEK, Fused Deposition Modeling (FDM), 3D printing, scanning probe microscopy, AFM, multifrequency AFM, viscoelastic, additive manufacturing

## Abstract

Polyether ether ketone (PEEK) is a high-performance thermoplastic polymer known for its unique combination of properties that make it suitable for a wide range of applications. Despite significant advancements in the characterization of PEEK, its high melting point (343 °C) presents challenges in both sample preparation and post-processing treatments such as annealing. Due to the high melting temperature of PEEK, there is a large change in temperature that occurs during the deposition of each layer during the print, causing a lack of strong adhesion between each filament layer. Therefore, annealing becomes a necessary post-processing step to ensure strong bonding within the parts. Hence, there is a need to establish precise post-processing parameters to enhance the material’s structural integrity and performance. This study aims to characterize PEEK at both the nanoscale and the macroscale by utilizing Atomic Force Microscopy (AFM) and mechanical testing methods such as tensile and three-point bending tests. AFM imaging, which offers high-resolution surface analysis, was used to assess PEEK’s surface morphology before and after annealing, providing insights into roughness, mechanical properties, and structural integrity at the nanoscale. Tensile and bending tests evaluated PEEK’s mechanical performance under macroscale conditions. Microscale AFM revealed that annealing at higher temperatures and for longer durations enhances polymer chain mobility. This promotes structural reorganization, recrystallization, and a reduction in surface roughness. These findings correlate to the macroscale properties where the tensile strength of the sample with the longest annealing duration and highest temperature increased 6.0 MPa from the sample that was not annealed. Three-point bending tests showed a 16 MPa increase from the unannealed sample to the sample annealed at 360 °C for 6 h. The findings from this research will help optimize post-processing parameters for PEEK, improving material quality while contributing to the broader understanding of its surface and mechanical properties. This work provides valuable data for future studies and applications involving high-performance polymers, especially within engineering and biomedical fields.

## 1. Introduction

Polyether ether ketone (PEEK) is a high-performance thermoplastic polymer known for its unique combination of properties that make it suitable for a wide range of applications. Its widespread use in aerospace, medical, automotive, and electrical sectors is a testament to its versatility [1,2,3,4,5]. PEEK’s unique attributes—such as its remarkable thermal stability, chemical resistance, high tensile strength, stiffness, fatigue resistance, dimensional stability, biocompatibility for medical applications, excellent electrical insulation, and lightweight nature—enable it to perform reliably in the most demanding environments.

Despite these advantages, processing and characterizing PEEK remain challenging due to its high melting point (343 °C). Proper thermal management is essential not only during fabrication but also in post-processing treatments such as annealing, which significantly influences PEEK’s crystallinity, mechanical properties, and surface morphology. Annealing plays a crucial role in optimizing the material’s performance by reducing internal stress, improving crystallinity, and enhancing mechanical properties. However, achieving precise control over annealing conditions and understanding their effects at both the microscale and macroscale require further study.

High-resolution Atomic Force Microscopy (AFM) is a valuable tool for analyzing PEEK’s surface morphology at the nanoscale. This includes the identification of nanoscale features, surface roughness, and height distributions [6,7]. The instrument uses a cantilever with a sharp tip that interacts with the sample surface. A laser focused on the cantilever’s back reflects onto a position-sensitive photodiode (PSPD), detecting deflections caused by surface interactions. A piezoelectric scanner moves the tip in a raster pattern, creating high-resolution surface images [8,9].

Contact mode is one of the primary methods used in AFM to characterize surfaces at the nanoscale. In contact mode, the tip maintains continuous contact with the surface, operating in the repulsive regime of the force–distance curve [10]. A feedback loop adjusts the cantilever height to maintain constant force. While this mode provides topographical information, the compressive and shear forces can deform soft samples, potentially reducing image resolution [11].

In tapping mode, the probe intermittently contacts the surface in a tapping motion where the cantilever oscillates at its resonant frequency, which reduces tip–sample interaction times. During operation, the tip momentarily interacts with the surface during each oscillation cycle, “tapping” the sample. This intermittent contact reduces lateral forces that could damage the sample or wear the tip. The tip experiences attractive and repulsive forces, governed by van der Waals interactions and the Pauli exclusion principle [10]. A feedback loop adjusts the vertical position of the cantilever to maintain a constant oscillation amplitude [12]. Larger oscillation amplitudes tend to favor the repulsive regime and are more typically used for imaging harder surfaces and smaller oscillation amplitudes keep the tip primarily in the attractive regime, ideal for softer samples. Tapping mode introduces phase imaging where the phase lag of the tip relative to the excitation signal is monitored and recorded while the feedback loop keeps the amplitude at a fixed value [13]. This is important to the phase image because a larger phase lag corresponds to materials with higher energy dissipation or viscoelasticity. Stiffer areas tend to produce smaller phase shifts and softer areas often produce larger phase shifts. Therefore, when comparing the tapping mode to the contact mode, the tapping mode provides more insights into the material’s mechanical properties as well as the surface topography.

Bimodal mode leverages two distinct resonance frequencies of the cantilever eigenmodes to enhance AFM capabilities. The first eigenmode works off a feedback loop that keeps the cantilever at a constant oscillation amplitude. The second eigenmode operates without direct feedback and independently responds to tip–sample interactions. These interactions occur at a smaller amplitude and shorter timescale than the first eigenmode, significantly increasing sensitivity to material variations [14]. The higher eigenmode with higher sensitivity to tip–sample interactions allows bimodal AFM to extract detailed nanoscale information about stiffness, viscoelasticity, and energy dissipation [14,15].

Through AFM characterization, the surface morphology and mechanical properties of PEEK at the microscale including stiffness, tensile strength, and surface characterization will be observed. Therefore, microscale analysis can provide information on how the annealing time and temperature affect the crystallinity and molecular structure of PEEK.

The macroscale analysis, including tensile and flexural property evaluations, is compared with the microscale analysis to identify correlations and solidify results. Tensile and flexural parameters were identified in previous studies as some of the most essential parameters that significantly influence the quality of 3D-printed parts [16]. Linking microscale and macroscale properties is important because it creates a comprehensive understanding of how a material’s small-scale features influence its overall performance. Understanding these relationships is vital for optimizing PEEK’s performance, especially for applications requiring precise material behavior, including 3D printing.

For this study, tensile and flexural samples were fabricated using Fused Deposition Modeling (FDM) 3D printing, which is an additive manufacturing (AM) technique that has become increasingly popular due to its capacity for both mass production and high customization [17]. The process begins by feeding a thermoplastic filament (PEEK) into a heated nozzle that melts the material. The molten filament is then precisely extruded onto a build platform according to a 3D model, which is first sliced into thin layers using specialized software. As each layer cools, it bonds to the layer below, gradually constructing the final object. FDM printing requires careful control of parameters like temperature, print speed, and layer height to ensure high-quality results and reduce issues such as warping or weak layer adhesion.

With growing interest in additive manufacturing for high-performance polymers, optimizing PEEK’s properties through post-processing techniques has become increasingly important. In fact, the global market of high-performance polymers for AM was forecast to grow by 24% between 2023 and 2028, with an estimated market of USD 362 million in 5 years [18]. However, ensuring the mechanical reliability of 3D-printed PEEK components remains a challenge, particularly due to its high melting point and the need for precise thermal control. By refining post-processing techniques such as annealing, this study aims to enhance PEEK’s mechanical properties and overall performance, ultimately contributing to the advancement of high-performance AM applications.

The primary objective of this study is to investigate the effects of annealing on PEEK’s microstructural and mechanical properties. Through microscale AFM characterization and macroscale mechanical performance, this research aims to contribute to the broader understanding of post-processing optimization for high-performance polymers. The findings will provide guidelines for improving post-processing strategies, which in turn could enhance the mechanical reliability of 3D-printed PEEK components. These insights will be valuable for engineering and biomedical applications where precision and material performance are critical.

In recent years, research on 3D-printed PEEK has grown primarily due to the material’s potential to combine the benefits of additive manufacturing with the mechanical properties of PEEK. Achieving high-quality 3D-printed PEEK parts with excellent mechanical properties poses distinct challenges particularly related to the material’s high melting point (343 °C) and the need for precise thermal control during printing. Researchers face significant challenges when 3D printing PEEK, primarily due to the high temperatures required to process the material. The printer must reach and maintain extremely high temperatures to ensure the PEEK filament flows smoothly and can be deposited properly. Additionally, preventing PEEK from clogging the nozzle is a critical challenge, as its high melting point and tendency to solidify quickly can cause blockages. Achieving precise thermal control throughout the printing process is essential to avoid these issues and ensure consistent, high-quality prints [19].

While existing studies provide valuable insights into the FDM 3D printing of PEEK, there remains a notable gap in the literature regarding comprehensive analyses of the processing–structure–property relationship of PEEK. In most cases, research is based on the limited processing conditions and mechanical properties of PEEK. While some studies chose only thermal processing conditions, others only chose parameters such as layer thickness, raster angle, or printing speed. For instance, Wu et al. [20] only explored the influence of layer thickness and raster angle on the mechanical properties of FDM-printed PEEK. In another study, Wang et al. [21] only looked at the influence of PEEK’s properties when printing with two different 3D printers. Xiaoyong et al. [22] examined the impact of filling ratio, build plate, and chamber temperature on the tensile strength of PEEK. On the other hand, Lannunziata et al. [23] studied the effect of PEEK properties on various infill properties and two different heating techniques. Vaezi and Yang [24] only investigated the effect of different thermal parameters like nozzle, bedplate, and chamber temperature on small-sized PEEK parts’ mechanical properties. Therefore, existing studies in the literature do not thoroughly investigate the influence of different processing parameters on the microscale and macroscale properties of 3D-printed PEEK parts in a single report.

Although existing studies offer crucial insights into AFM analysis techniques on PEEK and annealing, the literature lacks a thorough investigation connecting 3D-printed PEEK samples with various post-processing techniques analyzed through AFM. In most cases, research limited AFM analysis of thermal processing conditions after 3D printing. While some studies only chose to look at annealed samples, others chose to only look at 3D-printed samples with little to no post-processing techniques. For example, Kurtaran [25] analyzed the effect of annealing on Al-doped ZnO thin films with surface characterization through AFM. Similarly, Singh et al. [26] studied the influence of thermal annealing on microstructural features of graphene oxide using the AFM. On the other hand, Badeghaish et al. [27] examined the impact of high-temperature annealing on PEEK thin films exposed to hydrochloric acid (HCl) using AFM. Qian et al. [28] looked at PEEK which was heated with a thermal probe under the AFM only utilizing contact mode to gain information on surface topography. For 3D-printed PEEK, Karunanithi et al. [7] looked at how different layer heights and printing speeds affected PEEK under the AFM. Therefore, existing studies in the literature do not thoroughly investigate the influence of different post-processing parameters on the microscale and macroscale properties of 3D-printed PEEK parts in a single report.

The effects of post-processing treatments, such as annealing, remain an area of ongoing research. Understanding how annealing influences PEEK’s microstructural and mechanical properties will help bridge this gap by providing extensive nanoscale and macroscale data, ultimately optimizing its performance in various applications. The comparison with existing studies will validate the results and potentially reveal new aspects of PEEK’s behavior at the nanoscale and macroscale after annealing conditions.

## 2. Materials and Methods

### 2.1. Material

**Figure 1:**
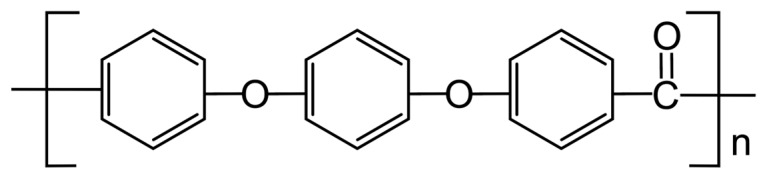
Chemical structure of polyether ether ketone (PEEK) [29].

As a member of the polyaryletherketone (PAEK) family, PEEK is defined by its semi-crystalline structure, featuring aromatic rings that provide rigidity, ether linkages that are flexible bonds that impart some elasticity, and ketone groups that contribute to the polymer’s high melting point (343 °C), which is drawn in Figure 1 [29]. This semi-crystalline structure allows PEEK to be a versatile polymer with a wide range of applications.

The PEEK powder used for AFM samples is grade 450G PEEK powder purchased from Victrex [30] with material properties outlined in Table 1.

The PEEK filament used for FDM 3D printing is the KetaSpire^®^ MS NT1 AM polyetheretherketone filament [35] with material properties outlined in Table 2.

### 2.2. Three-Dimensional Printing Process

**Figure 2:**
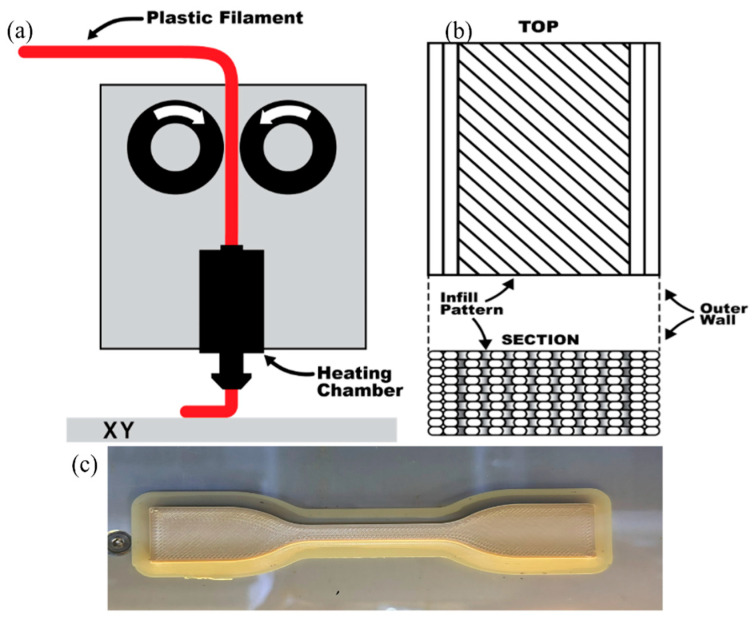
(**a**) The diagram of a standard extrusion process for Fused Deposition Modeling (FDM) 3D printing. The plastic filament is fed through a motor into a heated chamber then through the nozzle and onto the build platform. (**b**) This diagram represents the extrusion pattern showing 2 walls and solid infill at a 45° raster angle. (**c**) ASTM D638 3D-printed PEEK tensile sample with 5 mm brim.

**Figure 3:**
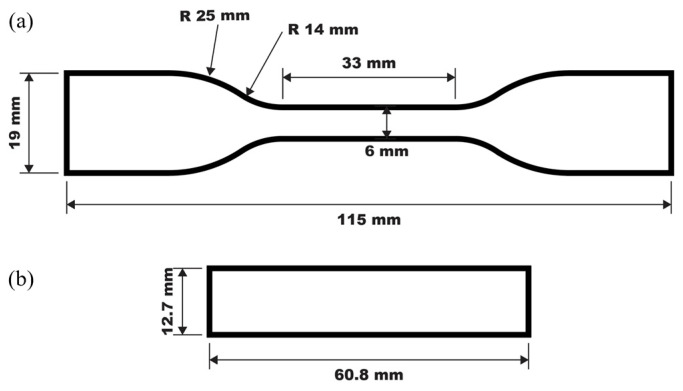
(**a**) ASTM D638 tensile sample dimensions, (**b**) ASTM D790 three-point bending sample dimensions.

PEEK samples are 3D-printed using a Fused Deposition Modeling (FDM) 3D printer called CreatBot PEEK-300 [39]. Figure 2a illustrates the diagram of a standard extrusion process for FDM 3D printing where the plastic filament (PEEK) is fed through a motor into a heated chamber then through the nozzle and onto the build platform. The nozzle temperature is 470 °C, the bed of the printer is 170 °C and the chamber is 120 °C to account for PEEK’s high melting temperature. The nozzle size used was 0.4 mm and the layer height was 0.2 mm for all samples, which were selected based on common commercially available FDM nozzle diameters for a balance of print speed and quality. The samples were printed in the x–y orientation. The sample was printed with 100% infill density in a rectilinear pattern with 2 perimeter walls and a solid infill at a 45° raster angle, which is shown in Figure 2b. Figure 2c is the ASTM D638 3D-printed tensile sample with a 5 mm brim that was removed and filed to clean the edges of the sample for post-processing. The dimensions of each sample for macroscale testing are listed in Figure 3 where Figure 3a represents the ASTM D638 tensile sample dimensions and Figure 3b represents the ASTM D790 three-point bending sample dimensions.

### 2.3. Annealing

**Figure 4:**
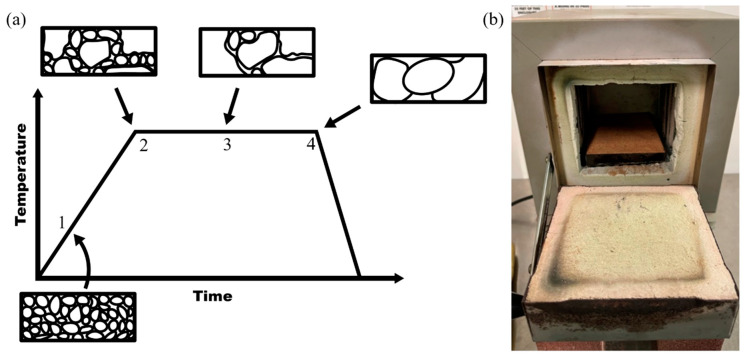
(**a**) Diagram of the progression of grain size and structure during annealing. (**b**) Thermolyne 1400 Furnace system used to anneal samples.

It is important to note that the annealing process that is being discussed for 3D-printed PEEK parts is different from the traditional annealing process. In this process, in addition to rearrangement of polymer structure at micro- and nanoscale as represented in Figure 4a, the annealing will help bond individual layers together as well. This is the reason that it is recommended to go above the melting temperature. However, the exact temperature and time duration are not provided for 3D-printed parts in the literature. The annealing time and temperature will influence factors such as crystallinity and mechanical properties [40]. Figure 4a illustrates the progression of grain size and structure during annealing and Figure 4b is the Thermolyne 1400 furnace system used to anneal samples. At the beginning of the annealing process (stage 1), the grains are small, irregular, and poorly connected where the material has lower tensile strength and thermal stability. As the temperature rises at stage 2, the chains begin to relax, reducing the internal stress, and smaller grains merge signaling the beginning of recrystallization. At stage 3, the grain growth accelerates during the isothermal hold. Existing crystalline regions grow as molecular chains align and pack more efficiently into more ordered regions. This enhances mechanical and thermal properties as the degree of crystallinity increases. At stage 4, the grains rearrange into large, well-defined grains, reaching maximum crystallinity. The structure becomes highly ordered and the material achieves increased thermal, mechanical, and chemical properties. The samples are then rapidly cooled at room temperature in a constant temperature room. Initially, the grains are small and irregular, but as the annealing time and temperature increase, the grain size progressively enlarges, forming a more regular and ordered structure. The effect of annealing on mechanical properties improves mechanical properties due to the dimensional stability [41]. Figure 4 aligns with PEEK’s need to undergo controlled heating and cooling to optimize its molecular structure for the chains to rearrange and crystalize to achieve optimized crystalline properties.

The samples were post-processed using the annealing technique at the five conditions listed in Table 3. The 330 °C and 360 °C temperatures were chosen to investigate how PEEK performs before and after its melting point (343 °C). Table 4 displays the samples after annealing for tensile and flexural testing along with AFM samples. The sample size for this study was three samples for each set.

### 2.4. Microscale Characterization Using AFM

The microscale characterization of PEEK was performed using AFM where the 1.0 cm powder samples were analyzed. The PEEK powder samples were annealed at the conditions outlined in Table 3.

**Figure 5:**
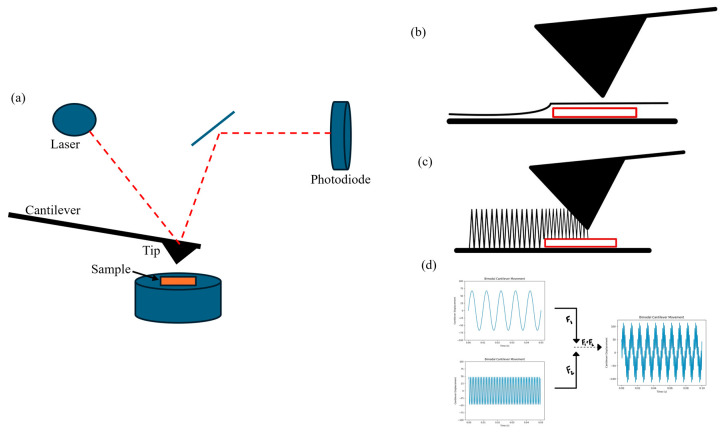
(**a**) Overview of the Atomic Force Microscopy (AFM) technique [43]. (**b**) Representation of contact mode on the AFM [44]. (**c**) Representation of tapping mode on the AFM [44]. (**d**) Representation of bimodal mode on the AFM [45].

AFM operates by scanning a sharp tip attached to a cantilever across a sample’s surface; as the cantilever responds to surface forces, a laser reflects off it onto a photodiode, creating high-resolution images of the surface, illustrated in Figure 5a. Each sample will start with imaging in contact mode, demonstrated in Figure 5b, in which the cantilever tip is held in constant contact with the sample surface to maintain constant cantilever deflection while scanning. For contact mode, a scanning probe with a 6–21 kHz resonance frequency range and spring constant 0.02–0.77 N/m was utilized. The scans were taken with decreasing scan sizes from 5 µm, 1 µm, 800 nm, 600 nm, to 400 nm and different areas of the sample surface were imaged to validate and verify the surface properties. Once contact mode images were saved, tapping mode was utilized. In tapping mode conveyed in Figure 5c, the probe intermittently contacts the surface in a tapping motion where the cantilever oscillates at its resonant frequency, and changes in oscillation amplitude and phase are used to create the image. Once tapping mode images were saved, bimodal mode was utilized. In bimodal mode exemplified in Figure 5d, the AFM cantilever operates by simultaneously exciting two eigenmodes. The first eigenmode is reserved for topographical information and is modulated using a feedback loop based on amplitude modulation. The second eigenmode is reserved for material properties and is run in an open loop. Therefore, it is common practice to extract topography images from the first eigenmode and phase images from the second eigenmode. For tapping and bimodal mode, a scanning probe with a 150 kHz resonance frequency and 9 N/m spring constant was utilized. Again, scans were taken with decreasing scan sizes from 5 µm, 1 µm, 800 nm, 600 nm, to 400 nm and different areas of the sample were imaged to validate and verify the surface properties.

A frequency sweep was performed to identify the resonance frequency of the AFM cantilever which involves scanning a range of frequencies to locate the cantilever’s resonance peak indicated by a graph that displays real-time feedback on the amplitude vs. frequency response. Fine adjustments are made to identify the optimal operating frequency where the cantilever responds. The gain is adjusted based on signal stability and response to eliminate excessive noise from defecting the scan. Once these parameters are set, the accuracy and resolution of the scans are assessed. The validation of the AFM scans confirms that the calibrated AFM system produces reliable and consistent data. To ensure accuracy, repeated AFM scans were conducted across different areas of the sample surface, verifying consistency throughout. Additionally, scans were performed at varying sizes to confirm that measurements were consistent at both the microscale and nanoscale. The AFM results were further validated by cross-referencing them with a standard reference sample of non-annealed PEEK, allowing for direct comparison of surface roughness and verifying the system’s reliability in detecting surface modifications due to annealing.

AFM analysis also derives the root mean square (RMS), which is a statistical measure used to quantify the variations in surface height across a given area [46]. RMS roughness provides a single value that represents the overall roughness of a surface in nanometers and is calculated by Equation (1) for a surface with height values z_i_ at N points where $\bar{z}$ is the mean surface height.

(1) $$\mathrm{RMS}=\sqrt{\frac{1}{N}\sum {\left(z_{i}-\bar{z}\right)}^{2}}$$

### 2.5. X-Ray Diffraction and Fast Fourier Transformation Process

**Figure 6:**
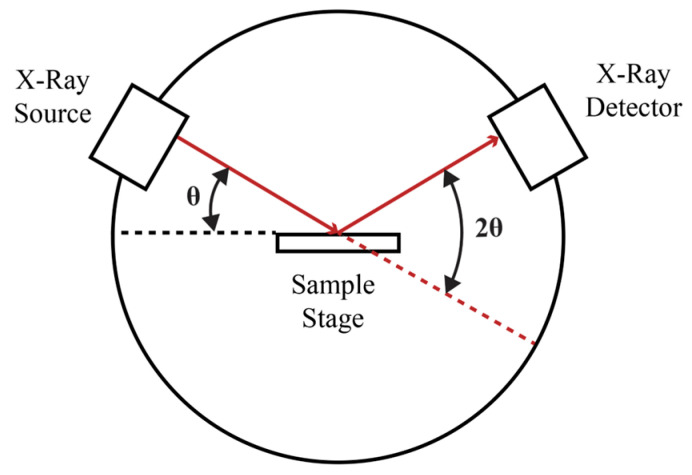
Overview of the X-ray diffraction process [47].

As seen in Figure 6, a powder X-ray diffractometer consists of an X-ray source, a sample stage, and a detector. The X-ray is focused on the sample at some angle θ, while the detector opposite the source reads the intensity of the X-ray it receives at 2θ away from the source path. The incident angle is then increased over time while the detector angle always remains 2θ above the source path. The diffractometer records the intensity versus 2θ angle as the X-ray beam scans across the angular range and a detector collects the scattered X-rays and converts them into an electronic signal [47].

The XRD data are then converted to Fast Fourier Transformation (FFT), which provides an analysis of periodic structures within the material. Since XRD data reflect the regular spacing of crystal planes, FFT can convert this spatial information into frequency information, revealing the periodicities more clearly. Peaks in the FFT output correspond to these periodic structures, allowing for detailed analysis of the crystalline arrangement. This conversion requires Bragg’s law, which describes the relationship between the spacing of atomic planes in a crystal and the angles at which the X-rays will be most intensely reflected from those planes.

Equation (2) represents Bragg’s Law which converts the diffraction angle (2θ) into d-spacings that represent the distance between atomic layers in the crystal lattice. In this equation, n is the order of diffraction, *λ* is the wavelength of the X-rays, d is the distance between lattice planes, and θ is the angle of incidence.

(2) nλ=2dsinθ

Convert d-spacing (a spatial measure) to the spatial frequency, which is the reciprocal of d-spacing, where q is the scattering vector magnitude in Equation (3).

(3) $$q=\frac{1}{d}$$

This analysis is primarily dictated by Bragg’s Law, which relates the diffraction angle to the atomic plane spacing (d-spacing) within the crystal lattice. This relationship allows for a deeper understanding of the structural properties of crystalline materials.

### 2.6. Macroscale Mechanical Testing

**Figure 7:**
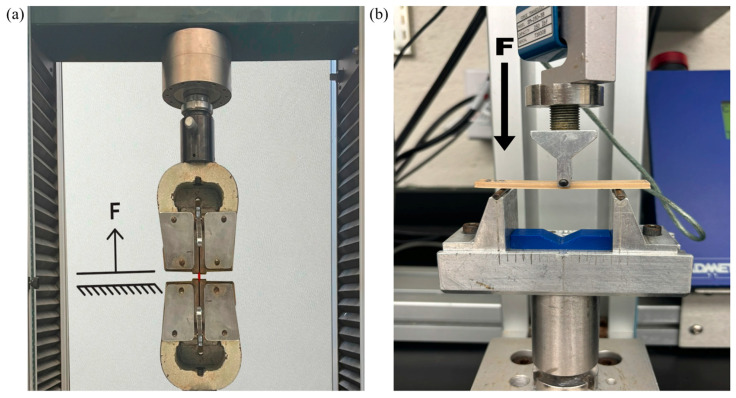
(**a**) Tinius Olsen testing apparatus used for ASTM D638 testing where the sample is pulled vertically, and the force is measured. (**b**) ADMET testing apparatus used for ASTM D790 testing where the force is applied and measured at the midpoint.

Tensile tests were performed on the Tinius Olsen shown in Figure 7a, which collected the force applied (F) and the change in position of the head, which is also the change in the length of the sample (ΔL). By knowing the initial cross-sectional area (A) and length (L_o_), the stress (σ) and strain (ε) at each data point can be determined using Equations (4) and (5), respectively.

(4) $$\sigma =\frac{F}{A}$$

(5) $$\varepsilon =\frac{\Delta L}{L_{o}}$$

By graphing the stress versus strain, the slope of the linear elastic region is Young’s modulus or modulus of electricity, and the maximum stress is the Ultimate Tensile Strength.

Three-point bending tests were conducted on the ADMET shown in Figure 7b, which collects the force applied (F) as well as the distance deflected. The flexural stress (σ_f_) can be determined for any load applied using Equation (6) based on the support span for the sample (L), width of the test beam (b), and thickness of the test beam (d).

(6) $$\sigma_{f}=\frac{3\mathrm{FL}}{2{\mathrm{bd}}^{2}}$$

The Ultimate Flexural Strength (UFS) is determined as the flexural stress at the maximum load applied. By determining the slope (m) of the linear elastic region of the force versus deflection plot, Equation (7) can be used to find the flexural modulus (E_f_) of the sample.

(7) $$E_{f}=\frac{L^{3}m}{4{\mathrm{bd}}^{3}}$$

For each set of samples, the average UTS and UFS were compared to determine the ideal annealing condition for PEEK materials post-printing.

## 3. Results and Discussion

### 3.1. Microscale Characterization Results

Topography AFM scans are represented in a mud color where the lightest regions are at the top of the scale bar and the darkest regions are at the bottom of the scale bar. Therefore, high-contrast images with bright/white spots next to dark/black spots represent large height inconsistencies that illustrate irregularity and roughness among the sample surface. In addition to height inconsistencies, height scans are also beneficial for visual analysis of the sample surface because they provide a direct, high-resolution representation of the topographical features. In addition to topography scans, the amplitude scans highlight differences in surface structures. The amplitude scans are represented as the grayscale color in all figures.

**Figure 8:**
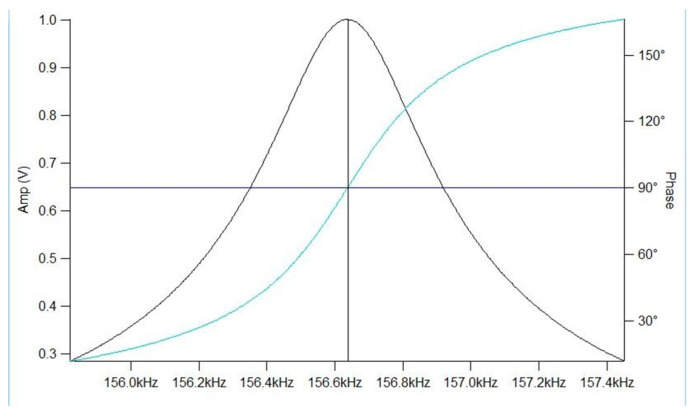
Amplitude and phase vs. excitation frequency [48].

Phase scans provide additional information about the material’s composition and mechanical behavior. The phase scans are represented as the inferno color in all figures. The phase AFM scan maps the phase shift of the cantilever’s oscillation as it interacts with the surface which depends on the material’s mechanical properties. Illustrated in Figure 8, areas of the sample that alter the local resonant conditions will shift the phase away from 90°, creating contrast in the AFM scan. Since AFM operates at or near resonance to maximize sensitivity, these phase shifts provide contrast in the AFM image, revealing variations in material properties. The scale bar to the right of the scans displays the degree of phase shift. A positive phase shift represents an attractive regime which is represented by the lighter areas on the phase scan. A negative phase shift represents the repulsive regime which is represented by the darker areas on the phase scan. When comparing phase images, the darker phase image represents stiffer properties. Phase scans also provide a visual representation of fine details, such as boundaries between regions with different material properties.

**Figure 9:**
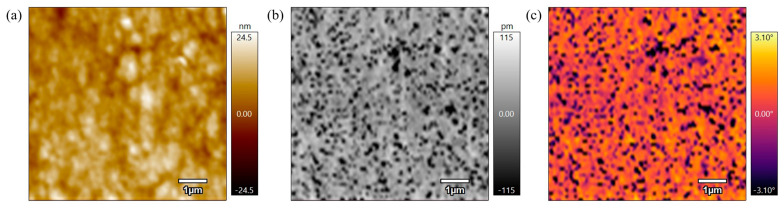
(**a**) 5 µm height AFM scan of PEEK powder not annealed. (**b**) 5 µm amplitude AFM scan of PEEK powder not annealed. (**c**) 5 µm phase AFM scan of PEEK powder not annealed.

Figure 9 represents the height, amplitude, and phase scans of a PEEK AFM sample that was not annealed to be used as a control. As seen in the height scan in Figure 9a, the surface of this sample has small, unorganized rough grains. The amplitude scan in Figure 9b shows that the surface structures have a grainy and rough appearance. The phase scan in Figure 9c suggests varying material properties along the surface because of the contrast in the image. These scans indicate that the unannealed sample surface is unorganized which suggests a lack of uniformity in the material’s microstructure.

**Figure 10:**
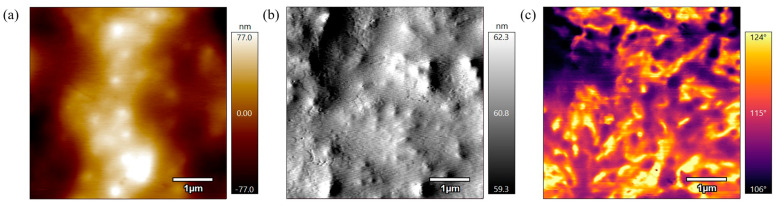
(**a**) 5 µm height AFM scan of PEEK annealed for 3 h at 330 °C. (**b**) 5 µm amplitude AFM scan of PEEK annealed for 3 h at 330 °C. (**c**) 5 µm phase AFM scan of PEEK annealed for 3 h at 330 °C.

Figure 10 represents the height, amplitude, and phase scans of the PEEK AFM sample annealed for 3 h at 330 °C. In Figure 10a the height scan shows the sample has a rough surface with high height discrepancies and appears coarse which indicates there are still small, unorganized grains. The amplitude scan in Figure 10b also illustrates the larger variations in height where there are random depressions and elevations along the surface. In Figure 10c the phase scan shows there is a contrast in phase values and irregularity across the surface. This indicates varying surface properties across the surface. When compared to the sample that was not annealed, there is a sign of the polymer starting to melt as the annealed sample surface scans are not as coarse. Although there is an improvement, the sample annealed at 330 °C for 3 h remains coarse and poorly defined, with no indication of a particle network.

**Figure 11:**
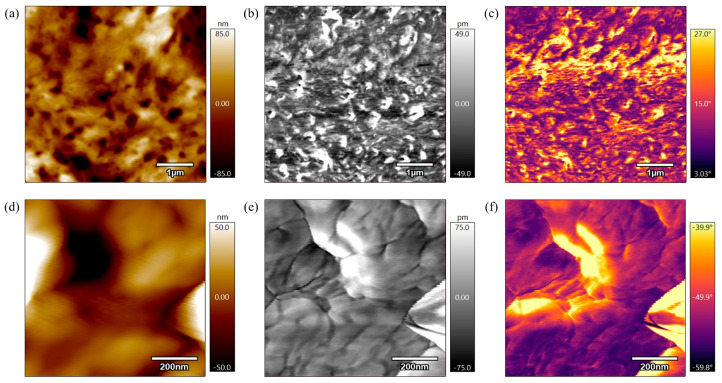
(**a**) 5 µm height AFM scan of PEEK annealed for 6 h at 330 °C. (**b**) 5 µm amplitude AFM scan of PEEK annealed for 6 h at 330 °C. (**c**) 5 µm phase AFM scan of PEEK annealed for 6 h at 330 °C. (**d**) 800 nm height AFM scan of PEEK annealed for 6 h at 330 °C. (**e**) 800 nm amplitude AFM scan of PEEK annealed for 6 h at 330 °C. (**f**) 800 nm phase AFM scan of PEEK annealed for 6 h at 330 °C.

Figure 11 represents the height, amplitude, and phase scans of the PEEK AFM sample annealed for 6 h at 330 °C. In Figure 11a,d, the height scans are not as grainy compared to the previous scans of not-annealed and annealed for 3 h at 330 °C samples, which indicates the sample continued to heat and the polymer chains became more relaxed. Figure 11b,e display the amplitude scans of PEEK annealed for 6 h at 330 °C. In Figure 11b, the 5 µm scan reveals a rough, heterogeneous surface with small, irregular structures distributed throughout the sample surface. On the other hand, in Figure 11e, the 800 nm scan reveals interconnected regions with clear boundaries which suggest crystalline domains. Figure 11c,f represent the phase scans of the PEEK sample annealed for 6 h at 330 °C where there is a lower phase shift compared to the previous samples indicating less variation in material properties on the surface. Specifically, Figure 11f visually displays the grain boundaries relaxing and merging leading to larger grains, more organized grains. Both the height, amplitude, and phase scans indicate some progress in the melting process, but the changes are not yet significant enough for recrystallization to occur.

**Figure 12:**
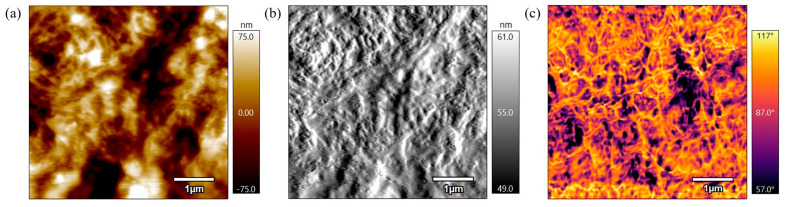
(**a**) 5 µm height AFM scan of PEEK annealed for 3 h at 360 °C. (**b**) 5 µm amplitude AFM scan of PEEK annealed for 3 h at 360 °C. (**c**) 5 µm phase AFM scan of PEEK annealed for 3 h at 360 °C.

Figure 12 represents the height, amplitude, and phase scans of the PEEK AFM sample annealed for 3 h at 360 °C. The height scan in Figure 12a illustrates the semicrystalline structure where the raised/brighter regions on the surface represent crystalline regions as they appear to be densely packed, and the lower regions are the amorphous regions. This is also apparent in the amplitude scan shown in Figure 11b where there are raised crystalline regions and lower amorphous regions visible. The phase scan in Figure 12c also visually distinguishes chains that are raised and in the process of melting on top of the amorphous regions that are lower. There is a significant contrast in phase values which suggest varying properties along the sample surface. Compared to the not-annealed sample and samples annealed at 330 °C, the polymer has begun to melt, and the scans visually show the crystalline and amorphous regions coexisting.

**Figure 13:**
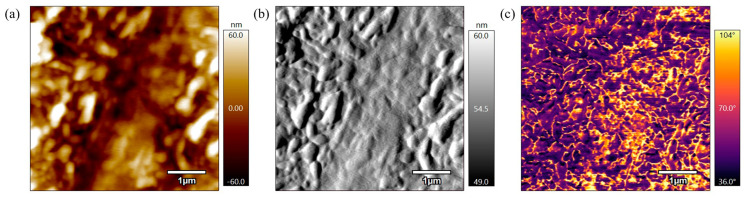
(**a**) 5 µm height AFM scan of PEEK annealed for 4 h at 360 °C. (**b**) 5 µm amplitude AFM scan of PEEK annealed for 4 h at 360 °C. (**c**) 5 µm phase AFM scan of PEEK annealed for 4 h at 360 °C.

Figure 13 represents the height, amplitude, and phase scans of the PEEK AFM sample annealed for 4 h at 360 °C. Figure 13a,b is comparable to Figure 12a,b, where the crystalline and amorphous regions are visible but in Figure 13 the sample has a smoother surface, indicating a more even surface due to the progression of the melting process. The phase scan in Figure 13c shows smaller grains beginning to merge into larger ones, with the network becoming tightly packed, indicating significant progress in the recrystallization process. In addition, this phase scan is darker compared to previous phase scans which indicates a stiffer material. The surface also shows improvement in the contrast of phase values where there is more uniformity of material properties along the surface. Overall, Figure 13 demonstrates the progression of the melting and recrystallization processes in the sample through reduced height discrepancies and the merging of smaller grains into a more tightly packed network.

**Figure 14:**
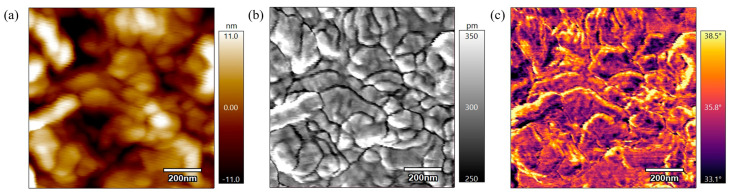
(**a**) 1 µm height AFM scan of PEEK annealed for 6 h at 360 °C. (**b**) 1 µm amplitude AFM scan of PEEK annealed for 6 h at 360 °C. (**c**) 1 µm phase AFM scan of PEEK annealed for 6 h at 360 °C.

Figure 14 represents the height, amplitude, and phase scans of the PEEK AFM sample annealed for 6 h at 360 °C. Based on these scans, the sample has undergone significant melting and structural reorganization. The grain boundaries are large, well-organized, and tightly packed, indicating that the thermal treatment of the samples led to the formation of a closely knit network of particles. The height scan in Figure 14a shows the lowest height discrepancy, indicating a smooth, interconnected surface and visually depicting a network of tightly packed particles. The amplitude scan in Figure 14b also illustrates the larger, interconnected grain boundaries. The phase scan in Figure 14c shows large, well-defined grain boundaries within a tight-knit network of particles. There is a low phase shift indicating the surface has consistent material properties. All scans indicate ongoing crystallization, with the surface properties becoming more consistent, resulting in a more stable and uniform surface.

**Figure 15:**
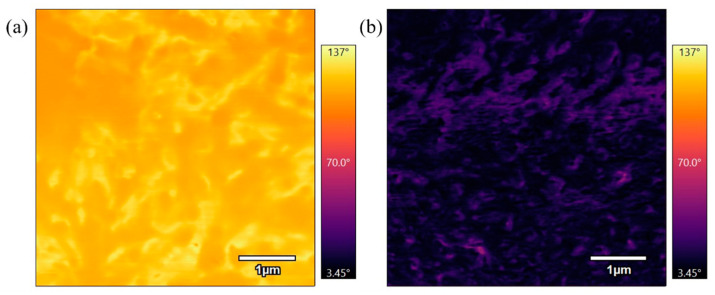
(**a**) 5 µm phase AFM scan of PEEK annealed for 3 h at 330 °C. (**b**) 5 µm phase AFM scan of PEEK annealed for 6 h at 330 °C.

Figure 15 compares phase scans of samples annealed at 330 °C for different durations. Figure 15a, which was annealed for 3 h, shows a bright phase image, while Figure 15b, annealed for 6 h, is a darker phase image. The difference in phase contrast suggests that the sample annealed for a longer duration has undergone more molecular rearrangement, resulting in a more rigid material. This indicates that longer annealing times promote greater crystallinity and stiffness in the material.

**Figure 16:**
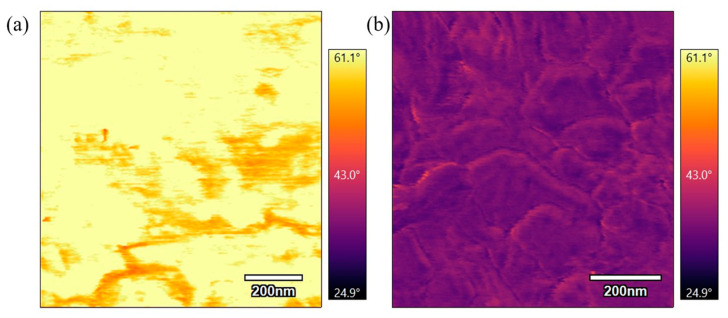
(**a**) 1 µm phase AFM scan of PEEK annealed for 3 h at 360 °C. (**b**) 1 µm phase AFM scan of PEEK annealed for 6 h at 360 °C.

Figure 16 compares phase scans of samples annealed at 360 °C for different durations. Figure 16a, which was annealed for 3 h, shows a bright phase scan, while Figure 16b, annealed for 6 h, is a darker phase scan. These scans are similar to the previous scans of samples annealed at 330 °C for varying durations where the longer duration at the sample temperature results in a stiffer surface. The results indicate that extended annealing times allow for increased polymer chain rearrangement and alignment, leading to a more rigid material.

**Figure 17:**
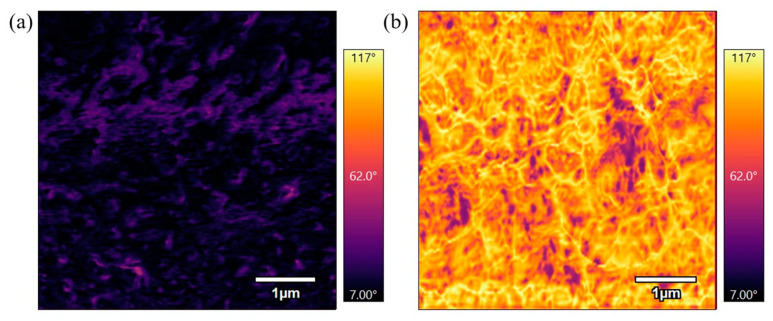
(**a**) 5 µm phase AFM scan of PEEK annealed for 6 h at 330 °C. (**b**) 5 µm phase AFM scan of PEEK annealed for 3 h at 360 °C.

Figure 17 compares phase scans of the lowest temperature with the longest duration and the highest temperature with the shortest duration. Figure 17a was annealed for 6 h at 330 °C and is a darker phase image while Figure 17b was annealed for 3 h at 360 °C and is a lighter phase image. The sample annealed for a longer duration at a lower temperature has higher stiffness compared to the sample annealed for a shorter duration at a higher temperature. This observation suggests that the extended annealing time at a lower temperature allowed for greater molecular rearrangement and increased stiffness. In contrast, the shorter duration at a higher temperature did not allow sufficient time for complete polymer chain alignment, resulting in a less stiff material. 

### 3.2. Correlation with Annealing Dosages

The comparison of all samples allows for further analysis of how the annealing technique changes the characteristics of the material surface. Samples annealed at 330 °C show gradual yet clear changes in surface morphology as the annealing time increases. The progression from coarse, unorganized grains to more relaxed polymer chains after 6 h indicates the beginning of structural reorganization, though complete recrystallization is not achieved. This suggests that extended annealing time at this temperature primarily facilitates chain relaxation and partial alignment without fully forming an interconnected crystalline network. In contrast, samples annealed at 360 °C exhibit more distinct and rapid melting and reorganization processes. Extended annealing times at this higher temperature led to a significant reduction in height elevations, the formation of larger grain boundaries, and the development of tightly packed particle networks. This suggests significant recrystallization and enhances structural uniformity to form a stable crystalline structure.

The comparisons between different durations and temperatures highlight the impact of annealing time and temperature on PEEK’s material behavior. Prolonged annealing at lower temperatures enhances polymer chain mobility, allowing gradual molecular rearrangement, which increases stiffness over time. Higher temperatures accelerate both melting and crystallization, promoting rapid structural reorganization and the formation of a tightly packed material network. These findings emphasize the importance of optimizing annealing conditions to attain specific material properties, such as smoother surface characteristics, improved flexibility, or greater structural stability. These findings on the annealing technique align with previous studies on other heating methods, such as scaffold molding, where higher temperatures led to increased crystallinity in PEEK [49]. This analysis emphasizes that annealing is a versatile tool for optimizing PEEK’s properties to meet specific engineering and industrial needs.

### 3.3. FFT Results

**Figure 18:**
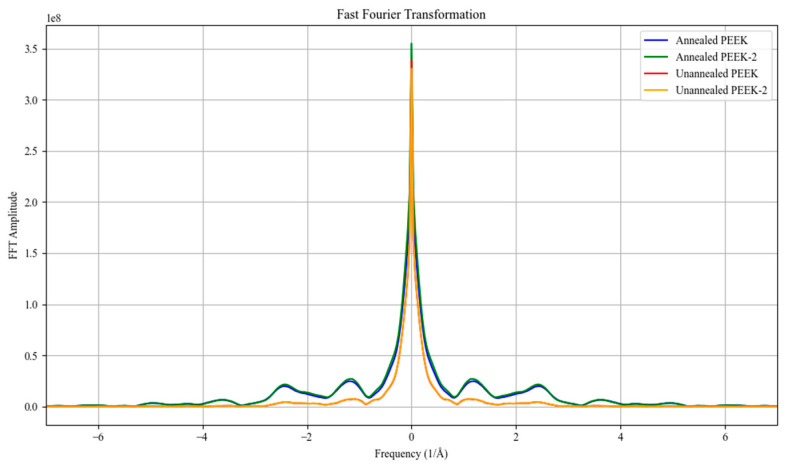
FFT results.

Figure 18 displays the Fast Fourier Transformation (FFT) results obtained from the X-ray diffraction data. FFT provides insights into the structural differences between annealed and unannealed PEEK. The graph illustrates the frequency–domain representation of the diffraction patterns, where the FFT amplitude reflects the intensity of periodic structural features within the material.

A distinct peak is observed at the central frequency for all samples. The sharpness and intensity of this peak indicate the presence of crystalline order, with the annealed samples (PEEK-330deg-3hr and PEEK-330deg-3hr-2) exhibiting slightly higher amplitudes compared to the unannealed samples (PEEK and PEEK-2). This suggests that annealing at 330 °C for 3 h enhances the crystalline content of PEEK, likely due to the increased molecular mobility during the thermal treatment, allowing the polymer chains to arrange into a more ordered structure.

Additionally, the presence of secondary frequency components, which appear as smaller peaks at non-central frequencies, provides information about the microstructural variations within the samples. The annealed samples show more pronounced secondary peaks than the unannealed ones, indicating a potential increase in crystalline domain size and improved long-range order. This observation aligns with previous studies that suggest annealing PEEK can refine its crystallinity by promoting better alignment of polymer chains [23].

The FFT analysis of the XRD data reveals that annealing PEEK significantly affects its structural characteristics. The increased intensity and sharpness of the primary peak, along with the more defined secondary peaks, suggest an improvement in crystallinity and long-range molecular ordering.

### 3.4. Macroscale Mechanical Properties

**Figure 19:**
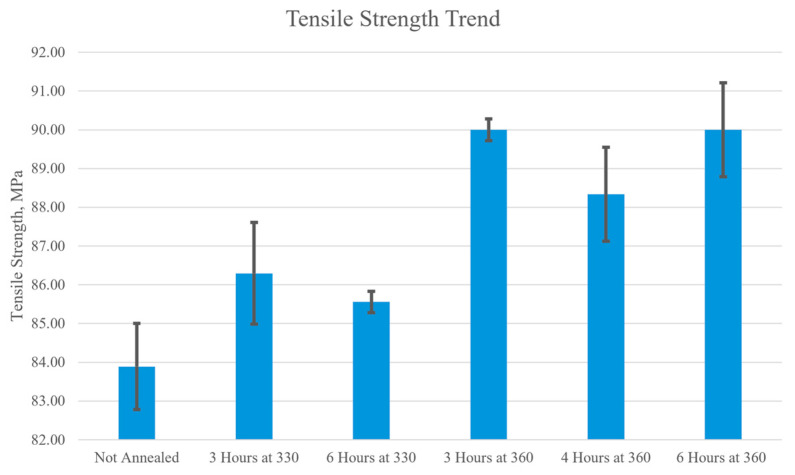
Bar graph of the tensile strength of each annealed PEEK sample.

The macroscale mechanical properties of PEEK were evaluated through tensile and three-point bending tests to assess its performance under different loading conditions. In tensile tests, the load condition is uniaxial tension, where pulling forces are applied along the axis of the specimen, causing it to stretch and elongate. The results of the tensile tests, presented in Figure 19, demonstrate a clear trend that annealing PEEK at higher temperatures and for longer durations increases its tensile strength. The tensile strength of the non-annealed sample was measured at 83.9 ± 2.22 MPa, while the sample annealed at 360 °C for 6 h exhibited a significantly higher tensile strength of 90.0 ± 2.42 MPa. This represents an increase of 6 MPa, highlighting the impact of prolonged annealing at elevated temperatures on improving the mechanical properties of PEEK. There was also an improvement when the annealing temperature increased from 330 °C to 360 °C. The samples annealed for 3 h at 330 °C were 86.3 ± 2.63 MPa and the samples annealed for 3 h at 360 °C were 90 ± 0.56 MPa. The samples annealed for 6 h at 330 °C were 85.6 ± 0.56 MPa and the samples annealed for 6 h at 360 °C were 90 ± 2.42 MPa. Therefore, the samples annealed for 3 h had a 3.7 MPa increase with the increase in temperature and the samples annealed for 6 h had a 4.4 MPa increase. Therefore, increasing the temperature of annealing increased the tensile strength of the PEEK material. These results suggest that higher annealing temperatures, especially with longer durations, increase the tensile strength of PEEK, which is consistent with the findings of previous studies [49,50,51].

**Figure 20:**
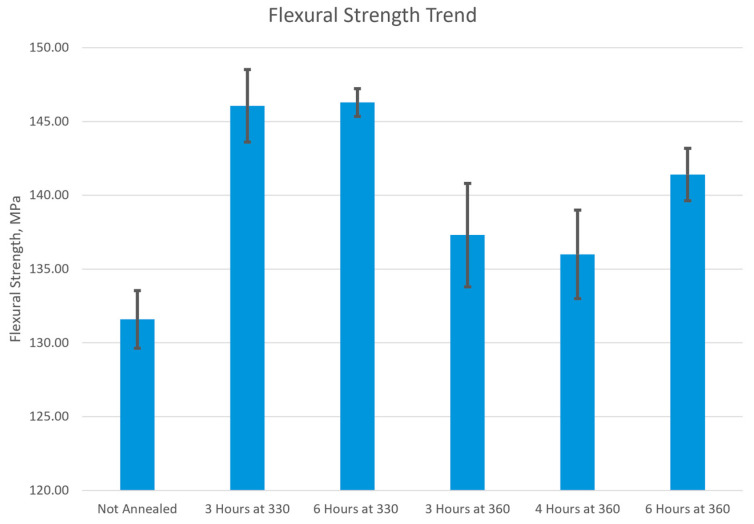
Bar graph of the ultimate flexural strength of each annealed PEEK sample.

In three-point bending tests the load condition involves bending under a combination of compressive and tensile stresses. The results of the three-point bending tests, illustrated in Figure 20, indicate the ultimate flexural strength of PEEK. When compared to the not-annealed sample with an ultimate flexural strength of 132 ± 1.8 MPa, all the annealed samples have increased in ultimate flexural strength. Therefore, an annealed flexural sample of PEEK had a higher ultimate flexural strength than a not-annealed sample, which was validated by previous studies [49,50,51]. Among the annealed samples, those treated at 330 °C had the highest flexural strength, reaching 146 MPa, while samples annealed at 360 °C showed lower values. Within the 360 °C samples, the longest duration, 6 h, had the highest ultimate flexural strength of 141 ± 3.5 MPa and the second highest was the shortest duration, 3 h, at 137 ± 7 MPa. The lowest ultimate flexural strength among the 360 °C samples and all annealed samples was observed at 4 h of annealing, with a strength of 136 ± 6 MPa. This conclusion for ultimate flexural strength was found in previous studies where the annealed samples exhibited a higher flexural strength when compared to a not-annealed sample [51].

### 3.5. Linking Microscale and Macroscale Properties

Understanding the relationship between microscale and macroscale properties is essential for optimizing the performance of PEEK in advanced applications. The microscale characteristics directly influence the material’s macroscale mechanical behavior. Smoother surfaces with large grain boundaries and a tightly interconnected network at the microscale should yield improved mechanical properties at the macroscale.

**Figure 21:**
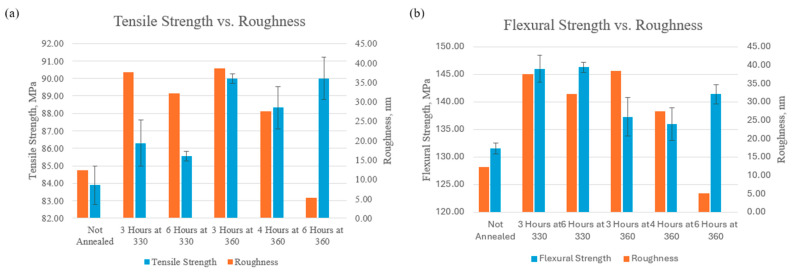
(**a**) Bar graph of the tensile strength compared to the roughness of the AFM height image. (**b**) Bar graph of the ultimate flexural strength compared to the roughness of the AFM height image.

Figure 21 compares the microscale characteristic of roughness to the macroscale mechanical analysis of tensile strength and ultimate flexural strength. The roughness of the surface topography starts lower with the not-annealed sample at 12.3 nm and then jumps to 37.7 nm when annealed for 3 h at 330 °C. The sample annealed for 6 h at 330 °C had a surface roughness of 32.2 nm and the sample annealed for 3 h at 360 °C had a surface roughness of 38.5 nm. These roughness values are relatively high and are due to the sample in the melting and recrystallization process. At a longer duration of 4 h at 360 °C, the roughness starts to drop as the recrystallization process progresses to 27.5 nm. Once the sample is annealed at the highest temperature, 360 °C, and longest duration, 6 h, the surface roughness drops to 5.21 nm, which means the polymer chains have relaxed. In Figure 21a, the surface roughness directly correlates to the tensile strength of annealed samples because the high-roughness samples have a lower tensile strength while the lower-roughness samples have a higher tensile strength. The not-annealed sample has a roughness of 12.3 nm and a tensile strength of 83.9 MPa while the sample annealed for 6 h at 360 °C has a roughness of 5.21 nm and a tensile strength of 90.0 MPa. Therefore, the surface roughness dropped by 7.06 nm, and the tensile strength increased by 6.1 MPa. In Figure 21b, the 330 °C samples have the highest flexural strength but also have the highest roughness value. When the roughness drops in the samples annealed for longer durations at 360 °C the flexural strength is still higher than the not-annealed sample but not as high as the samples annealed at 330 °C.

The results from the microscale and macroscale analyses reveal a connection between the surface morphology and mechanical properties of PEEK. Microscale AFM demonstrated that annealing at higher temperatures and longer durations enhances polymer chain mobility, promoting structural reorganization and recrystallization. This is because the samples annealed at 360 °C for extended durations formed larger, more tightly packed grain boundaries and smoother surfaces, which were visually determined from the AFM scans. On the macroscale, this reorganization of polymer chains is reflected in the improved tensile and flexural strength in the annealed samples. This indicates that the enhanced chain alignment and crystallinity at the microscale contributed to the increased strength at the macroscale. Overall, the microscale results provide valuable insights into the structural transformations at the surface of the material which in turn influences the macroscale mechanical performance.

## 4. Conclusions

This study investigated the effects of post-processing 3D-printed PEEK samples at the microscale and macroscale. The microscale analysis, conducted using AFM, revealed that annealing at higher temperatures and longer durations promotes polymer chain mobility, resulting in significant structural reorganization. This structural reorganization was represented by larger, more tightly packed grain boundaries and more uniform surface morphology in the annealed samples. The samples annealed for 6 h at 360 °C achieved the largest grain boundaries with interconnected particles, which indicates crystallization of the material. On the macroscale analysis, tensile testing showed that annealing at higher temperatures and longer durations improves the tensile strength of PEEK, with samples annealed at 360 °C for 6 h achieving the highest tensile strength. This can be attributed to the crystallization observed in microscale analysis where the tightly knit network of particles provided greater resistance to uniaxial tensile loading. The flexural strength results indicate that annealed samples demonstrated greater flexural strength compared to unannealed samples. Within the annealed samples, 330 °C samples had a higher flexural strength compared to 360 °C with a difference of 5 MPa. The results for both macroscale tests where the annealed samples have higher strengths align with the microscale results where annealed samples resulted in structural reorganization and strengthening.

The findings demonstrate the critical importance of tuning the properties of PEEK when annealing. The ability to control and optimize PEEK’s material structure and mechanical performance through adjustment of the annealing temperature and time provides a powerful method for customizing PEEK’s physical properties, surface uniformity, and mechanical stability. Future research could be carried out to explore additional annealing profiles and test PEEK in different environmental factors such as humidity and chemical exposure. The insights gained from this study could lead to the development of improved functionality and manufacturability for PEEK, enhancing its versatility and performance in various environments.

## Figures and Tables

**Table 1:** Material properties of Victrex [30] 450G PEEK powder.

Material Properties	Nominal Value	Unit	Test Method
Density	1.30	g/cm^3^	ISO 1183 [31]
Melting Temperature	343	°C	ISO 11357-2 [32]
Tensile Stress	98.0	MPa	ISO 527-2 [33]
Shore Hardness (Shore D, 23 °C)	84.5		ISO 868 [34]
Glass Transition Temperature Onset Midpoint	143 150	°C °C	ISO 11357-2

**Table 2:** Material properties of KetaSpire^®^ MS NT1 AM polyetheretherketone filament [35].

Material Properties	Nominal Value	Unit	Test Method
Density	1.29	g/cm^3^	ASTM D792 [36]
Melting Temperature	343	°C	ASTM D3418 [37]
Tensile Strength	85.0	MPa	ASTM D638 [38]
Filament Diameter	1.75	mm	

**Table 3:** Sample annealing conditions.

Annealing Conditions	Sample 1	Sample 2	Sample 3	Sample 4	Sample 5
Temperature (°C)	330	330	360	360	360
Time (hours)	3	6	3	4	6

**Table 4:** Testing samples.

	ASTM D638 Sample	ASTM D790 [42] Sample	AFM Sample
Unannealed	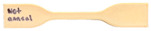	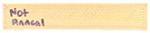	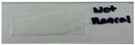
3 h at 330 °C	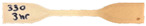	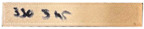	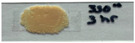
6 h at 330 °C	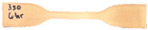	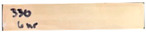	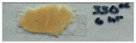
3 h at 360 °C	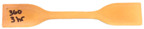	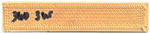	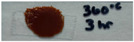
4 h at 360 °C	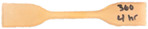	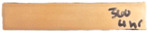	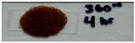
6 h at 360 °C	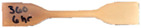	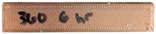	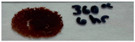

## Data Availability

The original contributions presented in this study are included in the article. Further inquiries can be directed to the corresponding author.
